# Baseline clinical characteristics predict overall survival in patients undergoing radioligand therapy with [^177^Lu]Lu-PSMA I&T during long-term follow-up

**DOI:** 10.1007/s00259-022-05853-2

**Published:** 2022-06-02

**Authors:** Philipp E. Hartrampf, Anna Katharina Seitz, Franz-Xaver Weinzierl, Sebastian E. Serfling, Andreas Schirbel, Steven P. Rowe, Hubert Kübler, Andreas K. Buck, Rudolf A. Werner

**Affiliations:** 1 Department of Nuclear Medicine, University Hospital Würzburg, Oberdürrbacherstraße 6, 97080 Würzburg, Germany; 2 Department of Urology and Paediatric Urology, University Hospital Würzburg, Oberdürrbacherstraße 6, 97080 Würzburg, Germany; 3 Department of Radiology and Radiological Science, The Russell H Morgan, Johns Hopkins University School of Medicine, 601 N Caroline Str, Baltimore, MD USA

**Keywords:** PSMA, Prostate cancer, [^177^Lu]Lu-PSMA I&T, Radioligand therapy, Overall survival, Prediction

## Abstract

**Background:**

Radioligand therapy (RLT) with ^177^Lu-labeled prostate-specific membrane antigen (PSMA) ligands is associated with prolonged overall survival (OS) in patients with advanced, metastatic castration-resistant prostate cancer (mCRPC). A substantial number of patients, however, are prone to treatment failure. We aimed to determine clinical baseline characteristics to predict OS in patients receiving [^177^Lu]Lu-PSMA I&T RLT in a long-term follow-up.

**Materials and methods:**

Ninety-two mCRPC patients treated with [^177^Lu]Lu-PSMA I&T with a follow-up of at least 18 months were retrospectively identified. Multivariable Cox regression analyses were performed for various baseline characteristics, including laboratory values, Gleason score, age, prior therapies, and time interval between initial diagnosis and first treatment cycle (*interval*_Diagnosis-RLT_, per 12 months). Cutoff values for significant predictors were determined using receiver operating characteristic (ROC) analysis. ROC-derived thresholds were then applied to Kaplan–Meier analyses.

**Results:**

Baseline C-reactive protein (CRP; hazard ratio [*HR*], 1.10, 95% *CI* 1.02–1.18; *P* = 0.01), lactate dehydrogenase (LDH; *HR*, 1.07, 95% *CI* 1.01–1.11; *P* = 0.01), aspartate aminotransferase (AST; *HR*, 1.16, 95% *CI* 1.06–1.26; *P* = 0.001), and *interval*_Diagnosis-RLT_ (*HR*, 0.95, 95% *CI* 0.91–0.99; *P* = 0.02) were identified as independent prognostic factors for OS. The following respective ROC-based thresholds were determined: CRP, 0.98 mg/dl (area under the curve [*AUC*], 0.80); LDH, 276.5 U/l (*AUC*, 0.83); AST, 26.95 U/l (*AUC*, 0.73); and *interval*_Diagnosis-RLT_, 43.5 months (*AUC*, 0.68; *P* < 0.01, respectively). Respective Kaplan–Meier analyses demonstrated a significantly longer median OS of patients with lower CRP, lower LDH, and lower AST, as well as prolonged *interval*_Diagnosis-RLT_ (*P* ≤ 0.01, respectively).

**Conclusion:**

In mCRPC patients treated with [^177^Lu]Lu-PSMA I&T, baseline CRP, LDH, AST, and time interval until RLT initiation (thereby reflecting a possible indicator for tumor aggressiveness) are independently associated with survival. Our findings are in line with previous findings on [^177^Lu]Lu-PSMA-617, and we believe that these clinical baseline characteristics may support the nuclear medicine specialist to identify long-term survivors.

## Introduction

Accounting for more than 375,000 deaths annually worldwide, metastatic castration-resistant prostate cancer (mCRPC) requires new treatment modalities [1]. Prostate-specific membrane antigen (PSMA)–targeted radioligand therapies (RLTs) using the β-emitting [^177^Lu]Lu-PSMA-617 led to increased overall survival (OS) when compared to standard of care [2] and superior biochemical response relative to chemotherapy with cabazitaxel [3]. Despite those promising results, a substantial fraction of patients do not respond to RLT in such a last-line setting, thereby emphasizing the need for reliable outcome predictors prior to treatment initiation. In this regard, multiple studies have suggested relevant prognostic factors for mCRPC patients receiving [^177^Lu]Lu-PSMA-617, including the presence of visceral metastases [4–7], elevated alkaline phosphatase (AP) [8, 9], or C-reactive protein (CRP) levels [10]. Further studies reported on the impact of prior chemotherapy [7, 11, 12], lactate dehydrogenase (LDH) [8–10, 13], aspartate aminotransferase (AST) [5, 14], and hemoglobin [5, 10] on survival for patients treated with [^177^Lu]Lu-PSMA-617.

The second most commonly administered radiotracer for PSMA RLT is [^177^Lu]Lu-PSMA I&T [15], and results of a currently recruiting prospective trial (NCT04647526) will be reported in due course [16]. Despite sharing the same PSMA-binding site, both radiotherapeutics exhibit substantial differences, in particular in regard to the utilized chelator linked to the peptide [17, 18]. Thus, outcome and prognostic parameters identified for [^177^Lu]Lu-PSMA-617 may not be necessarily applicable to patients treated with [^177^Lu]Lu-PSMA I&T. Using the latter compound, Heck et al. identified the presence of visceral metastasis and a rising LDH as independent predictors of OS [4], while Gafita et al. reported on the time since diagnosis of prostate cancer (PC) as a prognosticator for OS [19]. Supporting the notion that safety and efficacy profiles of both radioligands are less likely to be interchangeable, the current literature comprises only a limited number of approximately 100 subjects treated with [^177^Lu]Lu-PSMA I&T, which is in contrast to more than 900 reported patients with PC treated with [^177^Lu]Lu-PSMA-617 [15].

In this study that included 92 patients, we aimed to expand the current literature on [^177^Lu]Lu-PSMA I&T and to identify predictive baseline characteristics, focusing on long-term follow-up.

## Material and methods

### Patient cohort

**Fig. 1:**
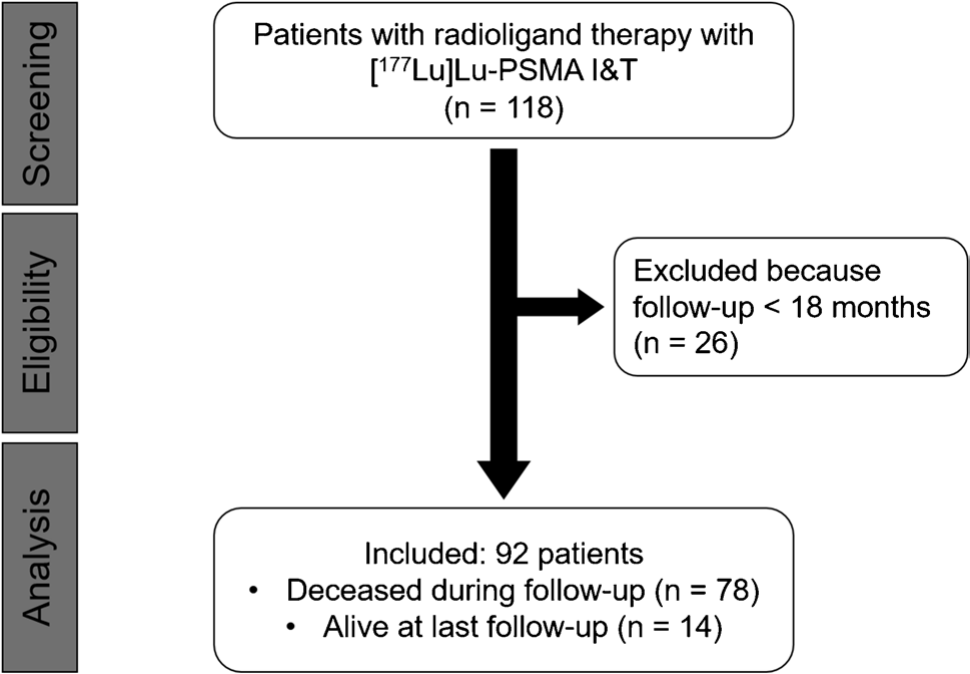
Flowchart of patients of excluded and included subjects

In this single-center study, we identified 118 men with mCRPC treated with [^177^Lu]Lu-PSMA I&T. Only patients with a follow-up period of at least 18 months after commencing the first treatment cycle were included. Exclusion criteria was a follow-up of less than 18 months (Fig. 1). All patients signed written informed consent. The local Ethics Committee waived the need for further approval due to the retrospective character of this investigation (waiver no. 20210422 04). Parts of this cohort have been reported in [20]. However, that previous analysis did not focus on identifying prognostic parameters for long-term survival.

### Pretherapy work-up

At the day of admission, pretherapeutic blood samples comprised serum chemistry (prostate-specific antigen (PSA) level, creatinine, LDH, AST, AP, CRP, and bilirubin) and routine hematology (leukocytes, hemoglobin, platelets). For blood draws, we utilized dipotassium ethylenediaminetetraacetic acid (EDTA) tubes (Sarstedt, Nuembrecht, Germany). Probes were then processed with an automated analyzer (Sysmex XN-9000, Kobe, Japan). For serum chemistry, serum-gel tubes were utilized (Sarstedt, Nuembrecht, Germany), followed by an analysis with a fully automated modular analyzer (Roche Cobas, Basel, Switzerland). Patient history was also retrieved from medical records.

### Treatment protocol

We conducted routine protocols for RLT. In this regard, [^177^Lu]Lu-PSMA I&T was synthesized as described elsewhere [20]. A total of 6.0 GBq of [^177^Lu]Lu-PSMA I&T was then administered every 42–56 days (maximum, 8 cycles per patient). If an individual exhibited markedly decreased renal or hematological function under RLT, the activity was reduced by approximately 20% 12/92 (13%) patients.

### Statistical analysis

Statistical analyses were performed using GraphPad Prism version 9.3.0 running on Windows (GraphPad Software, San Diego, CA, USA). We presented median and range in parentheses. OS was defined as the time interval of the day of the first cycle until day of death (presented as median). Since nearly all parameters were not normally distributed, comparisons between cohorts were performed using the Mann–Whitney *U* test. For comparison of dichotomous variables, we applied Fisher’s exact test. Uni- and multivariable Cox regressions were used to identify prognostic baseline parameters (including outlier correction). Cutoffs for survival prediction were determined by receiver operating characteristic (ROC) analysis and the Youden index for maximization of specificity and sensitivity. Kaplan–Meier curves and log-rank comparison were calculated to illustrate separation between responders and non-responders. We considered *P* < 0.05 statistically significant.

## Results

### Patients’ characteristics

A total of 92/118 (78.0%) patients were included in the final analysis (age, 71 years (52–90 years)). The median time interval between initial diagnosis and the 1st cycle of RLT (*interval*_Diagnosis-RLT_) was 75.5 (range, 9–364) months. The median initial Gleason score of all patients was 8 (5–10). Patients were treated with a median of three cycles (cumulative activity, 14.9 GBq).

### Baseline CRP, LDH, AST, and *interval*_Diagnosis-RLT_ are independent predictors of survival

Subdividing patients into those who were dead (n = 63) and those who were alive (n = 29) 18 months after initiation of RLT, no significant differences in previous treatment regimens were observed (*P* ≥ 0.1). Surviving subjects had a longer *interval*_Diagnosis-RLT_ (97 (25–364) vs. 64 (9–238) months, *P* = 0.006), and a higher number of RLT cycles (6 (2–9) vs. 2 (1–6), *P* < 0.001), with concomitant increased cumulative activity (33.4 (11.9–89.3) vs. 12.0 (4.9–36.7) GBq, *P* < 0.001). At baseline, survivors exhibited lower CRP (0.24 (0.04–3.30) vs. 1.47 (0.02–26.6) mg/dl, *P* < 0.001), lower LDH (209 (118–491) vs. 324 (173–1800) U/l, *P* < 0.001), lower AST (24.0 (15.5–62.0) vs. 31.8 (15.0–546.7) U/l, *P* < 0.001), and lower AP levels (89 (38–536) vs. 169 (31–5818) U/l, *P* < 0.001). Baseline hemoglobin, however, was substantially higher in surviving patients (11.9 (9.3–14.9) vs. 10.7 (6.0–16.1) g/dl, *P* = 0.002; Table 1).

**Table 1:** Baseline patient characteristics

	Entire cohort	Dead (*n* = 63)	Alive (*n* = 29)	*P*
***Clinical variables***				
Age at first cycle of PSMA RLT (years)	71 (52–90)	69 (52–90)	73 (55–89)	0.08
*interval*_Diagnosis-RLT_ (months)	75.5 (9–364)	64 (9–238)	97 (25–364)	0.006
Treatment cycles per patient	3 (1–9)	2 (1–6)	6 (2–9)	< 0.001
Cumulative activity (GBq)	14.9 (4.9–89.3)	12.0 (4.9–36.7)	33.4 (11.9–89.3)	< 0.001
Gleason score	8 (5–10)	8 (5–10)	8 (7–10)	0.91
***Baseline laboratory values***				
PSA (ng/ml)	192 (0.07–5000)	208 (0.07–5000)	93.8 (0.10–1640)	0.08
CRP (mg/dl)	0.82 (0.02–26.6)	1.47 (0.02–26.6)	0.24 (0.04–3.30)	< 0.001
LDH (37 °C U/l)	287.5 (118–1800)	324 (173–1800)	209 (118–491)	< 0.001
Hemoglobin (g/dl)	11.3 (6.0–16.1)	10.7 (6.0–16.1)	11.9 (9.3–14.9)	0.002
Creatinine (mg/dl)	0.92 (0.51–2.41)	0.89 (0.51–2.41)	1.02 (0.60–1.81)	0.24
Bilirubin (mg/dl)	0.4 (0.1–6.5)	0.4 (0.2–6.5)	0.4 (0.1–1.4)	0.74
AST (37 °C U/l)	28.1 (15.0–546.7)	31.8 (15.0–546.7)	24.0 (15.5–62.0)	< 0.001
AP (37 °C U/l)	131.5 (31.0–5818)	169 (31–5818)	89 (38–536)	< 0.001
***Prior treatments (%)***				
Radical prostatectomy	45.7	42.6	51.6	0.51
Primary radiation therapy to the prostate	15.2	11.5	22.6	0.22
Antihormonal treatment	100	100	100	1.0
Enzalutamide	69.6	72.1	64.5	0.48
Abiraterone	72.8	72.1	74.2	1.0
Chemotherapy	69.6	75.4	58.1	0.1

*interval*_*Diagnosis-RLT*_ time interval between initial diagnosis and 1st radioligand therapy, *PSA* prostate-specific antigen, *CRP* C-reactive protein, *LDH* lactate dehydrogenase, *AST* aspartate aminotransferase, *AP* alkaline phosphatase

On univariate analysis, CRP (*HR* 1.19, 95% *CI* 1.12–1.25; *P* < 0.001), LDH (1.10, 95% *CI* 1.06–1.14; *P* < 0.001), hemoglobin (0.72, 95% *CI* 0.62–0.83; *P* < 0.001), AST (1.20, 95% *CI* 1.12–1.30; *P* < 0.001), AP (1.07, 95% *CI* 1.04–1.11; *P* < 0.001), and bilirubin (1.55, 95% *CI* 1.01–2.16; *P* = 0.01) reached significance for OS. In addition, *interval*_Diagnosis-RLT_ (per 12 months, 0.94, 95% *CI* 0.90–0.98; *P* = 0.01) and previous radiation therapy (0.50, 95% *CI* 0.23–0.95; *P* < 0.05) were also significant predictors for OS (Table 2).

**Table 2:** Univariate and multivariable Cox regressions

	Univariate			Multivariable		
	*HR*	95% *CI*	*P*	*HR*	95% *CI*	*P*
CRP (per mg/dl)	1.19	1.12–1.25	< 0.0001	1.10	1.02–1.18	0.01
LDH (per 50 U/l)	1.10	1.06–1.14	< 0.0001	1.07	1.01–1.11	0.01
Hemoglobin (per g/dl)	0.72	0.62–0.83	< 0.0001	0.91	0.77–1.08	0.30
AST (per 10 U/l)	1.20	1.12–1.30	< 0.0001	1.16	1.06–1.26	0.001
AP 37 °C (per 50 U/l)	1.07	1.04–1.11	< 0.0001	1.03	0.98–1.07	0.30
*interval*_Diagnosis-RLT_*	0.94	0.90–0.98	0.01	0.95	0.91–0.99	0.02
Bilirubin (per mg/dl)	1.55	1.01–2.16	0.01	0.83	0.51–1.29	0.42
Primary RTx (yes)	0.50	0.23–0.95	0.05			
Chemotherapy (yes)	1.49	0.92–2.50	0.11	1.43	0.86–2.47	0.18
PSA (per µg/l)	1.00	0.99–1.00	0.13			
Age at 1st cycle (per year)	0.98	0.96–1.01	0.13			
Enzalutamide (yes)	1.39	0.85–2.37	0.21			
Reduced activity (yes)	1.28	0.64–2.32	0.45			
Gleason score	1.09	0.85–1.39	0.51			
RPE (yes)	0.88	0.55–1.38	0.57			
Abiraterone (yes)	1.09	0.66–1.86	0.75			
Creatinine (per mg/dl)	0.89	0.41–1.79	0.75			

*HR* hazard ratio, *CI* confidence interval, *CRP* C-reactive protein, *LDH* lactate dehydrogenase, *AST* aspartate aminotransferase, *AP* alkaline phosphatase, *interval*_*Diagnosis-RLT*_ time interval between initial diagnosis and 1st radioligand therapy, *RTx* radiation therapy, *PSA* prostate-specific antigen, *RPE* radical prostatectomy

^*^Per 12 months

Parameters reaching significance on univariate analysis were then included for multivariable Cox regression. Previous literature reported on the impact of previous chemotherapies on survival in subjects treated with [^177^Lu]Lu-PSMA-617 [7], and thus, this clinical variable was also included in our analysis. The following parameters remained significant prognosticators for OS: CRP (1.10, 95% *CI* 1.02–1.18; *P* = 0.01), LDH (1.07, 95% *CI* 1.01–1.11; *P* = 0.01), AST (1.16, 95% *CI* 1.06–1.26; *P* = 0.001), and *interval*_Diagnosis-RLT_ (0.95, 95% *CI* 0.91–0.99; *P* = 0.02), while all other variables did not reach significance (*P* > 0.1) (Table 2).

### Baseline CRP, LDH, AST, and *interval*_Diagnosis-RLT_ differentiate between responders vs. non-responders

**Fig. 2:**
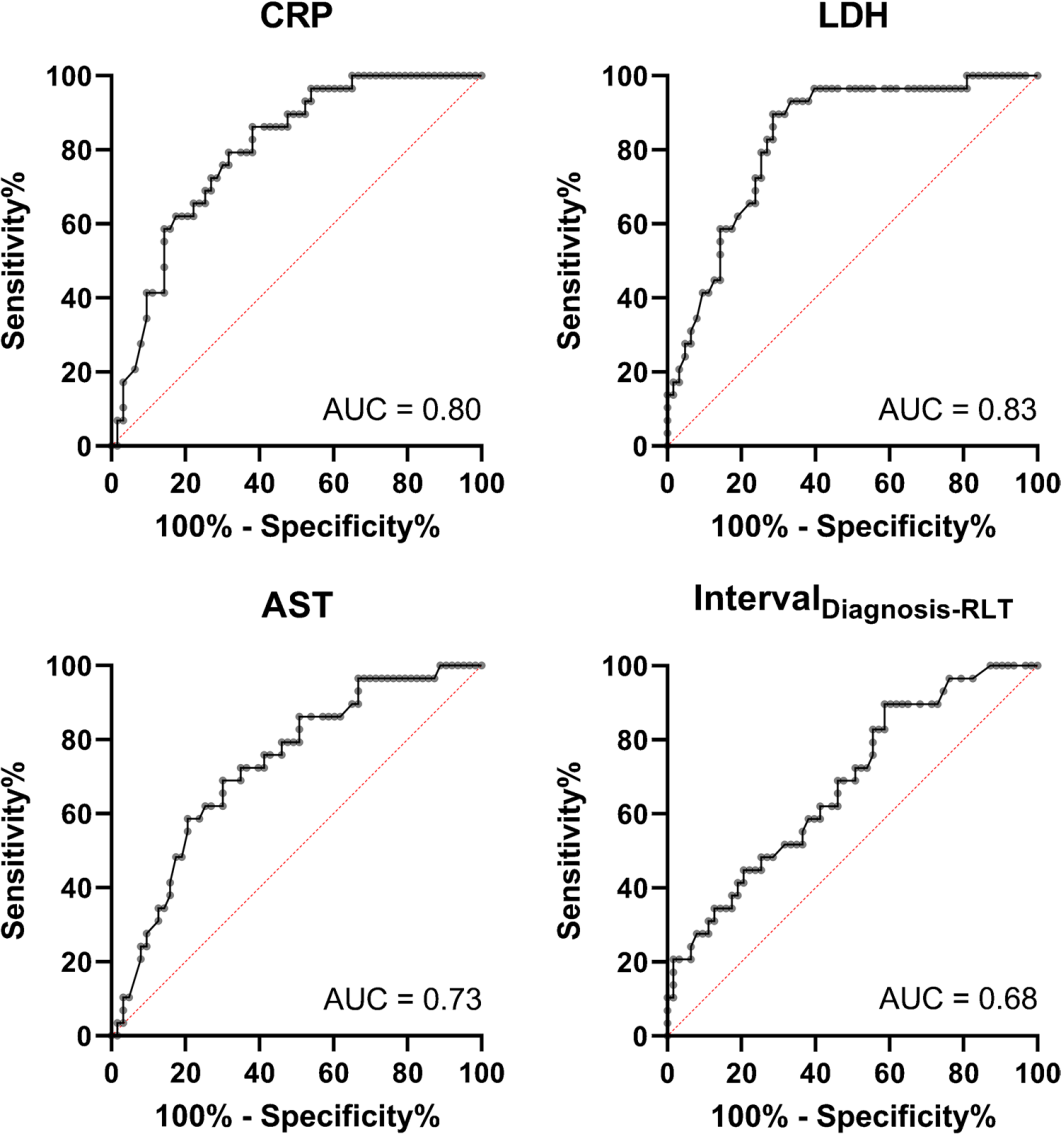
Receiver operating characteristics for baseline values of C-reactive protein (CRP), lactate dehydrogenase (LDH), aspartate aminotransferase (AST), and time interval between initial diagnosis and 1st radioligand therapy (*interval*_Diagnosis-RLT_). *AUC* area under the curve

**Fig. 3:**
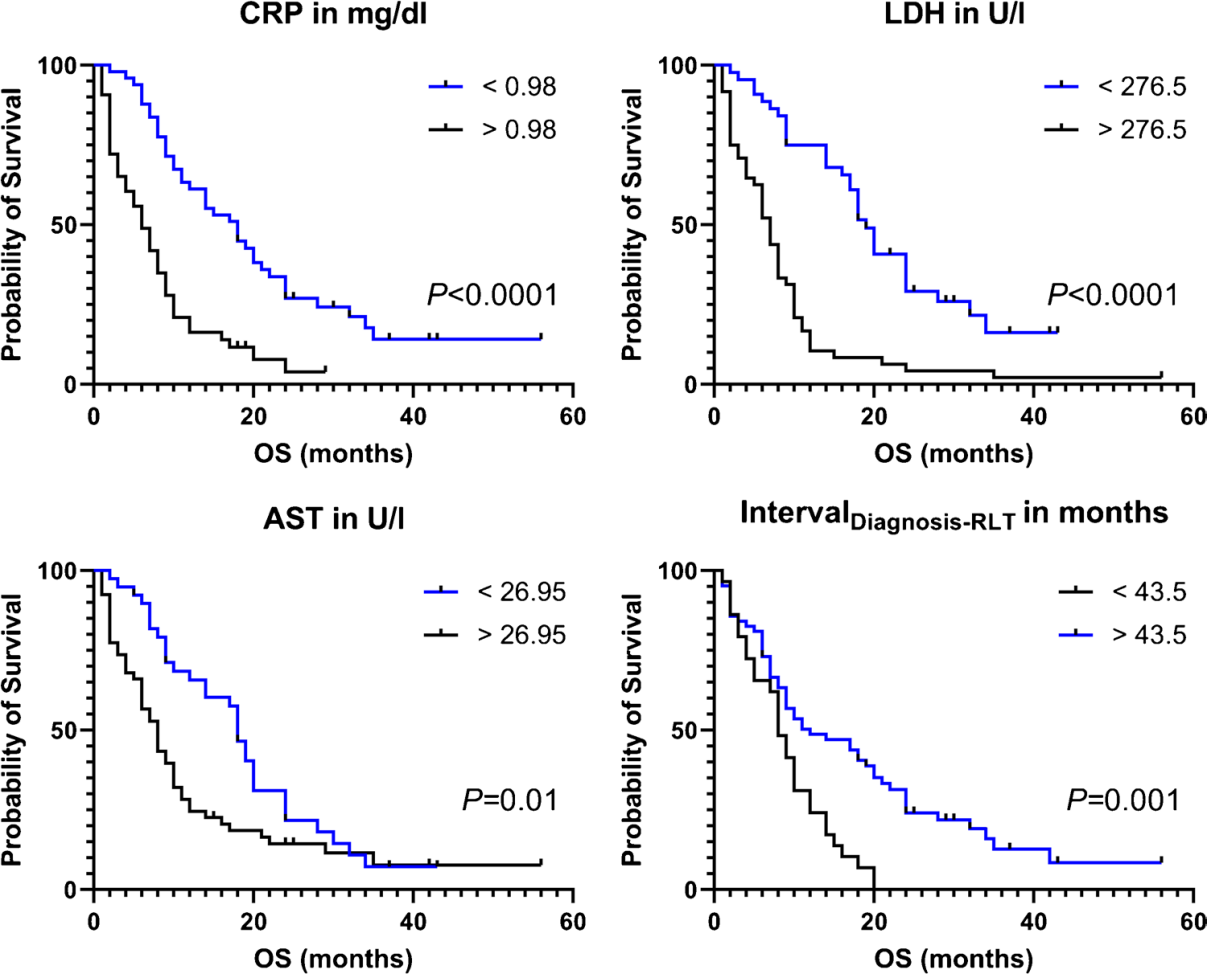
Kaplan–Meier curves for patients treated with radioligand therapy stratified into high- and low-risk patients using predefined cutoff values derived by receiver operating characteristics for C-reactive protein (CRP), lactate dehydrogenase (LDH), aspartate aminotransferase (AST), and time interval between initial diagnosis and 1st radioligand therapy (*interval*_Diagnosis-RLT_). Lower baseline CRP, LDH, and AST predicted a significantly longer median OS. In addition, the median OS of patients with a longer *interval*_Diagnosis-RLT_ was also prolonged

**Fig. 4:**
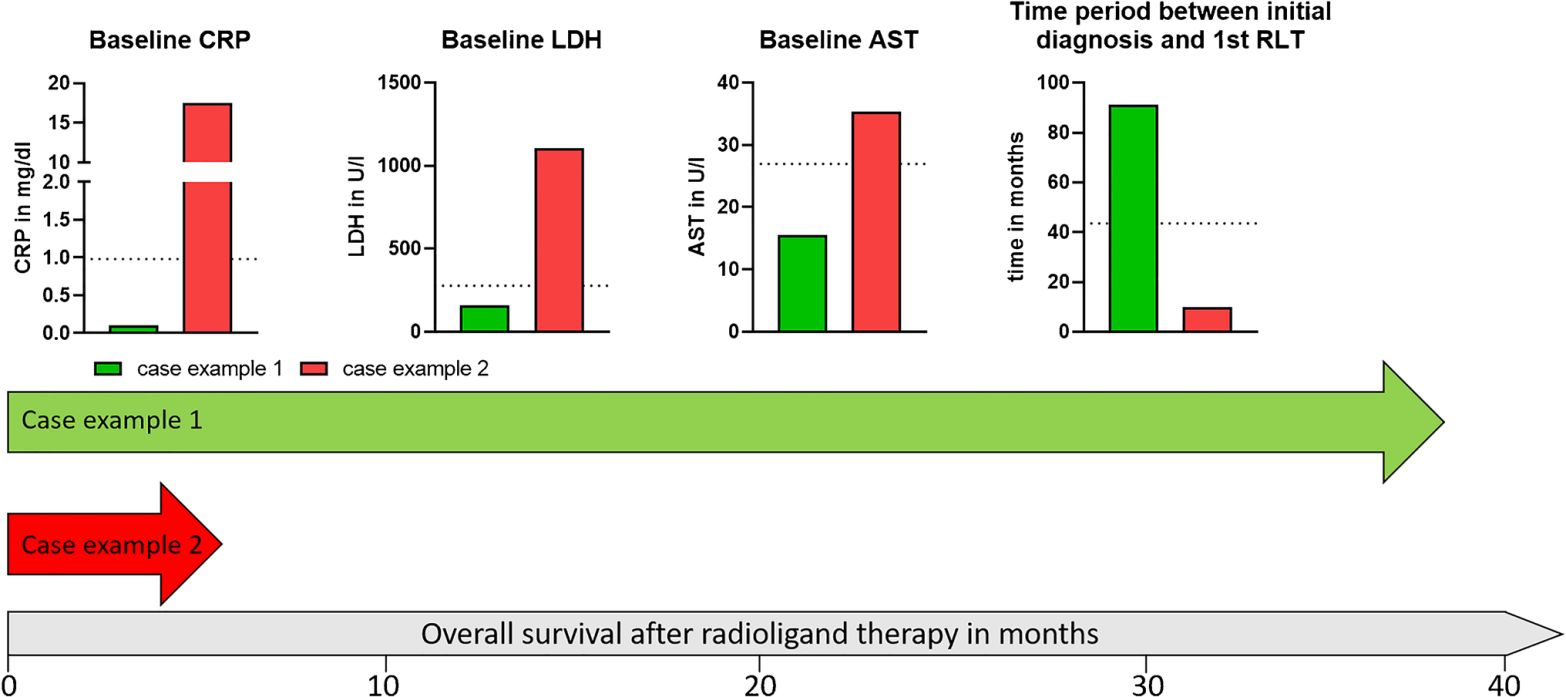
Examples of two patients with different outcomes after radioligand therapy with [.^177^Lu]Lu-PSMA I&T. The baseline values of the different parameters are presented in a bar chart. The dotted horizontal lines represent the (ROC-derived) cutoff values, and the large arrows visualize the overall survival

On ROC analysis, the following *AUC*s were determined: LDH, 0.83 (best cutoff, 276.5 U/l); CRP, 0.80 (best cutoff, 0.98 mg/dl); AST, 0.73 (best cutoff, 26.95 U/l; *P* < 0.001, respectively); and *interval*_Diagnosis-RLT_, 0.68 (best cutoff, 43.5 months, *P* = 0.006) (Fig. 2). Applying the derived cutoffs to Kaplan–Meier analyses, baseline CRP (18 vs. 6 months), LDH (19 vs. 7 months), AST (18 vs. 8 months), and *interval*_Diagnosis-RLT_ (12 vs. 8 months) separated between responders and non-responders (*P* ≤ 0.01, respectively) (Fig. 3). Figure 4 illustrates two cases of patients with different outcomes after RLT with [^177^Lu]Lu-PSMA I&T.

## Discussion

In this study that included 92 mCRPC patients treated with [^177^Lu]Lu-PSMA I&T, lower baseline CRP, LDH, and AST and longer *interval*_Diagnosis-RLT_ independently predicted longer OS. Using predefined cutoffs provided by ROC analysis, Kaplan–Meier analyses differentiated between low vs. high risks, with the most prominent segregation for LDH and CRP. Of note, the herein observed follow-up period of 18 months is almost twice as reported previously for patients being treated with [^177^Lu]Lu-PSMA I&T RLT [4]. Thus, the nuclear medicine practitioner can have certainty that those easily obtainable clinical characteristics can identify long-term survivors prior to therapy.

In recent years, a growing body of literature has reported on the favorable outcome of mCRPC patients treated with [^177^Lu]Lu-PSMA-617 in retrospective and prospective settings [2, 3, 15]. In this regard, various clinical baseline characteristics have been reported to identify patients that will most likely fail under RLT with this agent, e.g., CRP, LDH, liver enzymes, or AP [5, 9, 10, 14, 21]. However, as stated by Herrmann and coworkers, findings from previous studies using [^177^Lu]Lu-PSMA-617 have to be interpreted with caution in the context of RLT using [^177^Lu]Lu-PSMA I&T [22], mainly due to relevant differences in the chemical structure, i.e., using the different chelators DOTA and DOTAGA [17, 18]. Comparative studies on biodistribution and dosimetry showed longer half-lives in the tumor lesions for [^177^Lu]Lu-PSMA-617, but higher initial tumor uptake for [^177^Lu]Lu-PSMA I&T [23]. In addition, there is a significant imbalance in the current literature on the number of treated subjects with the latter agent (approximately *n* = 100) when compared to 617 (> 900) [15]. As such, further investigations focusing on outcome prediction with [^177^Lu]Lu-PSMA I&T are needed.

We therefore evaluated 92 subjects with the longest follow-up period reported to date and were able to identify comparable baseline characteristics having predictive potential relative to [^177^Lu]Lu-PSMA-617. In this regard, higher baseline CRP has already been described as a negative predictor of OS after RLT with [^177^Lu]Lu-PSMA-617 [5, 10, 14]. Although CRP is readily available in the clinic, this parameter is rather non-specific and can be substantially elevated due to inflammatory disease [24]. The influence of LDH on OS after RLT, however, is discussed controversially. For [^177^Lu]Lu-PSMA-617, elevated LDH has already been described as a negative predictor of OS [5], which was further corroborated by Ferdinandus et al. in an univariate analysis, identifying a comparable cutoff of 240.5 U/l when compared to our threshold (276.5 U/l) [9]. Of note, other univariate analyses failed to confirm LDH as a prognostic factor for OS [8, 10]. Also utilizing [^177^Lu]Lu-PSMA I&T, Heck et al. demonstrated that elevated LDH was a significant predictor of worse outcome [4], whereas the second most relevant study on this compound yielded no predictive potential of this parameter [19]. Similar to LDH, the predictive performance of AST is also a matter of debate. Ahmadzadehfar et al. reported that elevated AST (≥ 24 U/l) is linked to less favorable outcome for RLT with [^177^Lu]Lu-PSMA-617 [5], which is comparable to our ROC-derived cutoff of 26.95 U/l. Previous studies utilizing [^177^Lu]Lu-PSMA I&T did not investigate AST in the context of outcome prediction. Nonetheless, elevated baseline AST seems to be tightly linked to shorter survival regardless of which agent was applied.

Although reported for [^177^Lu]Lu-PSMA-617 in several studies [5, 8, 9, 11, 21], we could not confirm a prognostic ability of elevated baseline AP for OS after RLT with [^177^Lu]Lu-PSMA I&T, which is consistent with previous reports with this compound [4, 19]. As such, for this radiotracer, AP may play a less important role for outcome prediction when compared to [^177^Lu]Lu-PSMA-617.

Beyond laboratory values, longer *interval*_Diagnosis-RLT_ also demonstrated independent predictive potential for prolonged OS in our cohort. As a possible explanation, this baseline characteristic may represent tumor aggressiveness, as one may speculate that a longer interval between initial diagnosis and RLT is also linked to a more treatment-sensitive tumor biology. For instance, Barber et al. found an association between *interval*_Diagnosis-RLT_ and OS for taxane-naïve patients undergoing RLT with [^177^Lu]Lu-PSMA-617 in an univariate analysis [25], while Gafita et al. also reported that a longer *interval*_Diagnosis-RLT_ is associated with longer OS in men scheduled for [^177^Lu]Lu-PSMA I&T [19]. As such, this time frame may support a decision-making process to consider RLT for both radiotherapeutics.

The present study may provide in-depth results of patients that have been referred to our center in a real-world scenario with a focus on long-term follow-up. However, given the exploratory design of the study we cannot rule out a possible lack of power for testing the described associations. Therefore, we want to emphasize that the correlations found in our analysis have to be interpreted with caution as they not necessarily mean causation. Moreover, 12/92 patients received reduced activity in at least one cycle, but failed to reach significance in a univariate analysis (Table 2). Future studies may also control for this variable.

Further studies reported on the predictive value of image-based semi-quantification in the context of RLT [26, 27]. In the present study, however, we focused on clinical parameters, which are easy to obtain relative to the time-consuming nature of manual segmentation procedures of PSMA PET/CTs. Given the rather low number of reported subjects treated with [^177^Lu]Lu-PSMA I&T, we believe that the herein presented results are relevant for the practitioner [22]. Nonetheless, confirmatory clinical trials enrolling a substantially higher number of subjects are warranted, preferably also using PSMA-directed PET findings. In this regard, nomograms for patients treated with [^177^Lu]Lu-PSMA (including both 617 and I&T) have been established and also consider such PSMA-avid disease sites on molecular imaging. Of note, those risk charts have been provided to the community as online tools and, thus, can be easily tested for patients which had exclusively received [^177^Lu]Lu-PSMA I&T [6].

## Conclusion

In this study that included 92 patients with mCRPC treated with [^177^Lu]Lu-PSMA I&T, baseline laboratory values (CRP, LDH, and AST) and *interval*_Diagnosis-RLT_ (reflecting a possible indicator for tumor aggressiveness) were independent predictors of survival during long-term follow-up. Our findings corroborate with previous findings on [^177^Lu]Lu-PSMA-617 and we believe that these routinely available parameters may support the nuclear medicine physician to identify individuals that should be scheduled for RLT with [^177^Lu]Lu-PSMA I&T.

## Data Availability

The main data presented in this study are available in the article. Detailed information about the image analysis or the overall survivals of the subjects presented in this study is available on request from the corresponding author.
